# Treatment Considerations for the Management of Patients With Hormone Receptor–Positive Metastatic Breast Cancer

**Published:** 2014-09-01

**Authors:** Constance Visovsky

**Affiliations:** University of South Florida College of Nursing, Tampa, Florida

## Abstract

Breast cancer is among the most commonly diagnosed cancers in the United States. Despite treatment, 30% to 40% of women with early-stage or localized invasive breast cancer will eventually develop metastatic disease. Women with metastatic breast cancer (MBC) are living longer lives with the advent of new therapies. Currently, treatment for MBC can consist of a variety of approaches including chemotherapy, targeted therapy, and hormonal therapy, with disease-related, treatment-related, and patient-related factors guiding the selection and sequencing of these agents. In addition to controlling disease progression, strategies to improve or maintain quality of life are particularly important. For women with hormone receptor–positive disease, hormonal therapy is typically the first-line treatment of choice given the overall efficacy and favorable safety profiles of these agents; additional lines of other hormonal therapies are often administered upon disease progression. Other factors that must be considered by the practitioner to achieve optimal outcomes for the patient with MBC include the presence of comorbid illness and the educational, psychosocial, and supportive care needs of the patient.

In the United States, breast cancer is the most frequently diagnosed cancer among women (Siegel, Naishadham, & Jemal, 2012). Every year, more than 200,000 women are diagnosed with invasive breast cancer, and approximately 40,000 deaths are estimated to have occurred from the disease in 2012 (Siegel et al., 2012). Although newer, dose-intensive therapies have resulted in improved disease control, about 30% to 40% of women diagnosed with invasive breast cancer will eventually develop metastatic breast cancer (MBC; De Boer et al., 2012; Peto et al., 2012; Gonzalez-Angulo, Morales-Vasquez, & Hortobagyi, 2007), with approximately 4% to 6% of women presenting with metastatic disease at the time of the initial diagnosis (Cardoso, Fallowfield, Costa, Castiglione, & Senkus, 2011). Currently, more than 150,000 women are living with MBC in the United States, and this number is expected to increase (Mayer & Grober, 2006). The 5-year relative survival rate for women with localized (stages I through III) breast cancer is about 98%; however, the 5-year survival rate for women diagnosed with metastatic disease is significantly lower at 24% (National Cancer Institute, 2013).

Risk factors for the development of MBC include larger tumor size, positive lymph node status, stage T3/T4 disease (Barinoff et al., 2013), hormone receptor (HR)-negative status, and human epidermal growth factor receptor-2 (HER2) overexpression (Beslija et al., 2009). It is generally accepted that women with MBC represent a heterogeneous patient population with an unpredictable clinical course (Beslija et al., 2009). This review will examine the key clinical considerations involved in the optimal management of patients with HR-positive MBC.

For optimal management of MBC, patients need access to specialized oncology personnel who maintain focused expertise or knowledge of recommended treatment regimens, provide the appropriate care and monitoring associated with each regimen, and are capable of fostering personal relationships with their patients (Rchaidia, Dierckx deCasterle, DeBlaeser, & Gastmans, 2009; Halkett, Arbon, Scutter, & Borg, 2006). Women diagnosed with metastatic disease have reported significantly more emotional distress, impaired quality of life (QOL), and differing symptom burden as compared with women without metastasis, as advanced stages of cancer represent a greater risk of a shortened lifespan and ongoing cancer-related treatment to extend progression-free survival (Aranda et al., 2005).

As part of the oncology health-care team, advanced oncology practitioners play key roles in ensuring that patients with MBC receive optimal care through regular monitoring and evaluation of their status and treatment plan (Aranda et al., 2005; Chung & Carlson, 2003). Specific measures include monitoring for adverse events (AEs) of treatment, as well as potential complications associated with comorbid illness and providing rigorous supportive care management of disease- and treatment-related symptoms (Chung & Carlson, 2003). In addition, advanced oncology practitioners play pivotal roles in addressing the critically important educational and psychosocial needs of patients with MBC (Aranda et al., 2005).

## Treatment Goals for Patients With MBC

Although the main treatment goal for women diagnosed with early-stage breast cancer is to attain cure or prevent tumor recurrence, the primary goals of MBC treatment are to improve patient QOL (Aranda et al., 2005; Aranda et al., 2006) and extend survival (Chung & Carlson, 2003; Glück, Arteaga, & Osborne, 2011). Although treatment of MBC is not curative, there are many effective options to facilitate achievement of these goals. However, hormonal therapy remains the standard of care in the management of patients with HR-positive MBC, which is the focus of this review.

## Hormonal Therapy

Studies performed in the United States, Canada, and parts of northern Europe have reported that approximately 70% to 80% of patients with breast cancer have HR-positive disease, defined as estrogen receptor (ER)-positive and/or progesterone receptor (PR)-positive disease (Huang et al., 2005; Sandoval et al., 2013). Estrogen stimulates the normal growth and division of breast tissue cells by binding to the ER and inducing receptor dimerization, which prompts changes in gene expression and cell behavior (Harwood, 2004). However, in women with HR-positive breast cancer, the presence of estrogen can contribute to the growth of cancer (Osborne & Schiff, 2011). Hormonal therapy interferes with estrogen stimulation of breast cancer cells and is the prevailing standard of care for women with HR-positive breast cancer tumors in both the adjuvant and metastatic settings (National Comprehensive Cancer Network [NCCN], 2013).

A number of classes of hormonal therapies are available, and the mechanisms of action of the different classes are summarized in Table 1 (Harwood, 2004 NCCN, 2013; ProStrakan, 2011; Buzdar, Robertson, Eiermann, & Nabholtz, 2002; AstraZeneca Pharmaceuticals, 2004, 2012a, 2012b; Buzdar, 2008; Novartis Pharmaceuticals Corporation, 2011; Pfizer, 2012). Overall, the choice of hormonal therapy for women with MBC is based on several factors, including consideration of menopausal status and prior adjuvant hormonal therapy (Bernard-Marty, Cardoso, & Piccart, 2004; NCCN, 2013). Due to advancements in available hormonal therapy options and an increasing number of treatments, the role of advanced oncology practitioners in supporting and advising patients has become even more critical for guiding and educating patients about the potential immediate and long-standing side effects of these therapies (Harwood, 2004). The main classes of hormonal therapies commonly used in HR-positive MBC are discussed here.

**Table 1:**
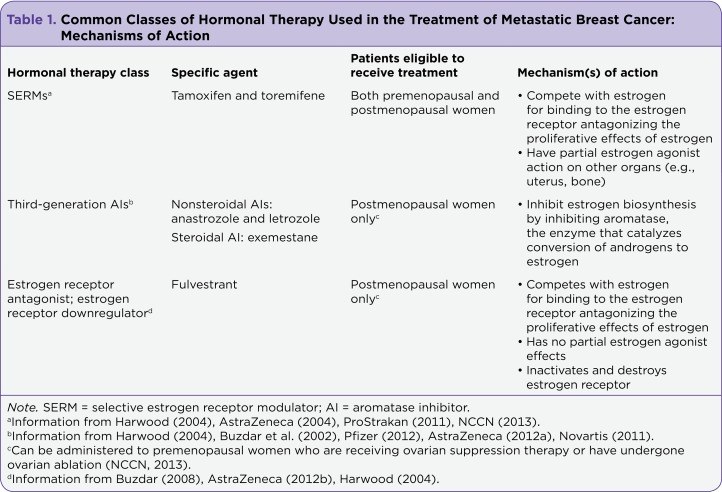
Common Classes of Hormonal Therapy Used in the Treatment of Metastatic Breast Cancer: Mechanisms of Action

## Selective Estrogen Receptor Modulators

For decades, selective estrogen receptor modulators (SERMs), such as tamoxifen, were the most widely used hormonal therapy for HR-positive breast cancer (Baumann & Castiglione-Gertsch, 2007). The efficacy and safety of tamoxifen have been demonstrated in numerous clinical studies in the setting of MBC, and it has been the comparator therapy for almost all other hormonal agents subsequently studied in this patient population (Bonneterre et al., 2000; Nabholtz et al., 2000; Mouridsen et al., 2003; Paridaens et al., 2008; Jordan & O’Malley, 2007). SERMs are considered first-line hormonal therapy options for women with HR-positive MBC, regardless of menopausal status (Harwood, 2004; NCCN, 2013). However, in premenopausal women with HR-positive MBC, a luteinizing hormone releasing hormone (LHRH) agonist for drug-induced ovarian suppression, or in some cases surgical removal of the ovaries, should be considered to deplete estrogen levels either prior to administration of hormonal therapy or subsequent to first-line hormonal therapy with tamoxifen (Cardoso et al., 2011; NCCN, 2013).

Mechanistically, the orally administered SERMs act as hormone receptor antagonists and compete with estrogen for available estrogen receptor binding sites to potentially halt or slow the progression of cancer growth. However, a partial agonist effect of SERMs is evident in some other organs, with advantageous effects seen on bone and negative effects observed in the endometrium, e.g., endometrial cancer (Table 1; Harwood, 2004; Buzdar, 1999).

## Aromatase Inhibitors

Although the ovaries are the main source of estrogen in premenopausal women, in postmenopausal women, estrogen is no longer produced in the ovaries (Harwood, 2004). However, in postmenopausal women, estrogen can still be produced ectopically by low levels of circulating androgens, which are subsequently converted to estrogen by the enzyme aromatase, present in many tissues, including breast cancer tumors (Harwood, 2004). Aromatase inhibitors (AIs) prevent the synthesis of estrogen through inhibition of aromatase; see Table 1 (Harwood, 2004; Buzdar et al., 2002; Pfizer, 2012; AstraZeneca Pharmaceuticals, 2012a; Novartis Pharmaceuticals Corporation, 2011). Because of their mode of action, AIs are incapable of completely blocking ovarian estrogen synthesis in premenopausal women. In addition, using AIs to suppress estrogen synthesis in premenopausal women causes increased compensatory estrogen production by the ovaries by way of a feedback loop through the pituitary gland. Consequently, AIs are only recommended for use as endocrine monotherapy in postmenopausal women (Buzdar et al., 2002; Harwood, 2004; Pfizer, 2012; AstraZeneca Pharmacueticals, 2012a; Novartis Pharmaceuticals Corporation, 2011). However, premenopausal women receiving ovarian suppression therapy or those who have undergone ovarian ablation are also candidates for AI therapy (Cardoso et al., 2011; NCCN, 2013; Barrios et al., 2012).

Two classes of AIs are currently available, and both are administered orally: nonsteroidal AIs (e.g., anastrozole [Arimidex] and letrozole [Femara]) and steroidal AIs (e.g., exemestane [Aromasin]). Nonsteroidal and steroidal AIs differ in their modes of inactivation of aromatase. Nonsteroidal AIs compete with the endogenous ligands androstenedione and testosterone for the active binding site of aromatase, where they form a strong but reversible bond, blocking both ligands and oxygen from aromatase. The steroidal AIs bind irreversibly to the active site through their metabolites, causing permanent inactivation of aromatase, even after elimination of the drug from the circulation (Buzdar et al., 2002).

The efficacy and safety of the AI agents have been well studied (Bonneterre et al., 2000; Nabholtz et al., 2000; Mouridsen et al., 2003; Paridaens et al., 2008), and they are considered first-line options for the treatment of patients with HR-positive breast cancer (NCCN, 2013).

## ER Downregulators

Similar to SERMs, ER downregulators bind to the ER and block estrogen binding. However, instead of simply competing with estrogen at ER binding sites, ER downregulators (e.g., fulvestrant [Faslodex]) completely inactivate and destroy ERs. Unlike SERMs, these drugs have no established partial agonist activity (Table 1; Buzdar, 2008; Harwood, 2004; AstraZeneca Pharmaceuticals, 2012b).

Like AIs, fulvestrant is indicated only for treatment of postmenopausal women but can also be administered to premenopausal women who are receiving ovarian suppression therapy or have undergone ovarian ablation and is considered a first-line option in the NCCN Breast Cancer Guidelines for patients with HR-positive disease (NCCN, 2013; AstraZeneca Pharmaceuticals, 2012b; Barrios et al., 2012). Fulvestrant is administered by intramuscular injection 3 times during month 1 of treatment and then monthly thereafter (AstraZeneca Pharmaceuticals, 2012b).

A recent study of the efficacy and safety of fulvestrant in patients with HR-positive MBC demonstrated a progression-free survival benefit with the 500-mg dose of fulvestrant compared with the 250-mg dose (Di Leo et al., 2010). Fulvestrant was well tolerated by patients in both treatment arms of this study, and the higher dose was not associated with increased toxicity. More recently, this study reported a 19% reduction in the risk of death with fulvestrant 500 mg vs. 250 mg (*p* = .016; Di Leo et al., 2012).

## Other Single-Agent Hormonal Therapies

The NCCN Guidelines on the management of patients with HR-positive MBC also include other single-agent hormonal therapy options (NCCN, 2013). These options include androgens (i.e., fluoxymesterone), progestins (i.e., megestrol acetate), and high-dose estrogen (i.e., ethinyl estradiol; NCCN, 2013). However, these agents are associated with increased toxicity profiles compared with the other hormonal therapies and are not typically used in the first-line setting (Howell & Howell, 2010; Buzdar et al., 1997). Nevertheless, they have been shown to be effective in some patients following disease progression on other hormonal therapies (Buzdar et al., 1997).

## Combination Hormonal Therapy and Hormonal Therapy Plus Targeted Biologic Treatments

Novel combinations of hormonal therapies as well as targeted therapies administered with a hormonal agent have been studied in the setting of MBC (Osborne & Schiff, 2011; Johnston, 2009). For example, there is some relatively recent evidence showing that the combination of anastrozole and fulvestrant in the first-line treatment of HR-positive MBC is more effective than anastrozole alone (Mehta et al., 2012). However, no difference in the efficacy of these regimens was observed in another similarly designed study (Bergh et al., 2012). One consideration when evaluating these results is that the dose of fulvestrant (250 mg) used in these studies was lower than currently recommended (500 mg; AstraZeneca Pharmaceuticals, 2012b).

In addition, therapies that target certain signaling pathways activated in cancer may also be used in combination with hormonal therapy (Glück et al., 2011). For example, activation of pathways involving the mammalian target of rapamycin (mTOR) has been linked to acquired resistance to hormonal therapy in ER-positive breast cancer (Osborne & Schiff, 2011; Baselga et al., 2012). Studies have shown that mTOR signaling results in phosphorylation and activation of the ER, causing estrogen-independent cell growth (Osborne & Schiff, 2011; Baselga et al., 2012). Hence, this finding provides a rationale for using therapies targeted to the mTOR pathway in patients with HR-positive MBC.

The oral mTOR inhibitor, everolimus (Afinitor), was approved for use in postmenopausal women with HER2-negative advanced HR-positive MBC in combination with exemestane (Pfizer, 2012), following the results of the Breast Cancer Trials of Oral Everolimus-2 (BOLERO-2) trial, which showed a 57% improvement in progression-free survival in the everolimus plus exemestane combination arm compared with single-agent exemestane in patients with HR-positive MBC (Baselga et al., 2012). However, high rates of stomatitis and infection were reported for patients in the combination arm (Novartis Pharmaceuticals Corporation, 2012).

Other combination regimens studied in patients with HR-positive MBC include single-agent AI therapy in combination with an anti-HER2 agent (e.g., lapatinib plus letrozole; trastuzumab plus anastrozole) for those with disease characterized as HER2-positive (Johnston et al., 2009; Kaufman et al., 2009). Although statistically significant increases in progression-free survival have been observed for patients receiving these combinations compared with those receiving single-agent AI therapy, no differences in overall survival have been seen. In addition, high rates of diarrhea, including grade 3/4 events, were observed in patients receiving the combination of lapatinib plus letrozole (Johnston et al., 2009).

## Benefits and Challenges Associated With Hormonal Therapy

One advantage of hormonal therapy is that many of these treatments are available as oral formulations, including tamoxifen, anastrozole, letrozole, and exemestane (AstraZeneca Pharmaceuticals, 2004, 2012a; Novartis Pharmaceuticals Corporation, 2011; Pfizer, 2012). Nevertheless, oral therapies, especially when administered over long periods, can be associated with treatment adherence and persistence problems (Fallowfield et al., 2006).

For example, in a study on the preferences of breast cancer patients for endocrine therapy according to drug formulation, approximately half the women surveyed admitted that they did not always take their current oral medication. Although more patients in this study reported a preference for oral vs. injected hormonal therapy, the benefit of improved therapy adherence was cited by 43% of patients who indicated a preference for injected therapy. For instance, intramuscular injections of fulvestrant, administered in a health-care setting on a once-monthly basis, can help ensure regular contact with a health-care provider.

Another important benefit of hormonal therapy is the possibility of continued clinical response following disease progression through sequential administration of different hormonal therapy agents, thereby delaying the use of chemotherapy (Barrios et al., 2012; NCCN, 2013). Evidence suggests that women with breast tumors that are clinically responsive to one type of hormonal therapy agent are likely to respond to another endocrine agent (Buzdar, 1999). However, the optimal sequence for hormonal therapy as single agents or in combination with other hormonal or targeted therapies is currently undefined and must be individualized (NCCN, 2013). Factors such as prior lines of hormonal therapies and the mechanisms of action of these agents are important considerations when selecting treatment.

There is evidence that hormonal therapy and chemotherapy have comparable survival benefits in patients with HR-positive disease (Beslija et al., 2009). However, hormonal therapy also tends to be well tolerated by patients with manageable side effects; hence, it can delay the need for chemotherapy.

Nevertheless, it is important to note that some hormonal agents may pose serious risks for individual patients. For example, tamoxifen is associated with an increased risk of uterine cancer and thromboembolic events (AstraZeneca Pharmaceuticals, 2004). Aromatase inhibitors may be associated with increased musculoskeletal symptoms, osteoporosis, and an increased risk of bone fractures (Lipton et al., 2003; Burstein, 2007). In addition to the impact of these side effects on overall health and QOL, there is evidence that the side-effect profile of hormonal therapy is likely to impact treatment adherence to oral agents (Murphy & Seidman, 2009). Furthermore, the high prevalence of comorbidities and their additive effects on daily functional status and well-being as it relates to QOL mandate the need for personalized treatment plans (Stewart et al., 1989).

For example, comorbidities, such as osteoporosis, should be considered before administering an AI (Abdulhaq & Geyer, 2008). The National Osteoporosis Foundation (NOF) recommends that all postmenopausal women be evaluated for osteoporosis risk to determine the need for additional diagnostic modalities such as bone mineral density (BMD) testing, which has been established as an excellent predictor of future fracture risk, and/or vertebral imaging. The NOF also recommends BMD testing for younger postmenopausal women with clinical risk factors for fracture. The use of AIs has been identified as a risk factor for osteoporosis and osteoporosis-related fractures (NOF, 2013).

One study of postmenopausal patients receiving AI therapy showed baseline musculoskeletal pain to be common in this population (Robidoux et al., 2011). Another example of a comorbid disorder or condition that can impact selection of hormonal therapy treatment in women with HR-positive MBC is a history of thromboembolic disorders in a patient considered for tamoxifen therapy (AstraZeneca Pharmaceuticals, 2004).

For patients receiving combination hormonal therapy or hormonal therapy administered in combination with targeted agents, the complexity of patient management strategies is likely to be increased, and defining an appropriate patient profile is important for obtaining optimal treatment efficacy. For example, the presence of heart disease in patients who are potential candidates for trastuzumab therapy and the side-effect profile of everolimus plus exemestane, especially for elderly patients, are important considerations (Genentech, 2010; Baselga et al., 2012; Pritchard et al., 2013).

## Supportive Care for Patients With HR-Positive MBC

Critical to the physical and mental health of patients with MBC is an environment in which they can communicate openly and regularly with their health-care providers. In such an environment, patients can be optimally monitored for disease- and treatment-related side effects. Supportive care, including frank discussions of hospice and palliative care options available to patients and families, can also be optimally administered.

In one study of patients with metastatic non–small cell lung cancer, the provision of early supportive care was associated with increased QOL and improved survival (Temel et al., 2010). The advanced oncology practitioner should engage in an ongoing dialog with patients and family members that incorporates plans for treatment as well as considerations for palliative care along the disease course.

Although a detailed discussion of the systemic supportive therapies used for patients with MBC is beyond the scope of this article, numerous options are available to address both treatment-related and disease-related side effects. For example, osteoclast inhibitors (e.g., bisphosphonates, denosumab) may decrease the risk of bone loss, particularly in patients receiving nonsteroidal AI therapy and are the standard of care in patients with bone metastases (Gralow et al., 2009; NCCN, 2013). For patients receiving hormonal therapy who experience musculoskeletal pain, a nonsteroidal anti-inflammatory drug (NSAID) may also be beneficial, and vitamin D supplementation has been suggested as an approach to address bone loss and musculoskeletal symptoms (Brueggemeier & Diaz-Cruz, 2006). An evaluation of the potential of cytotoxic agents to cause nausea and/or vomiting can allow for the effective administration of prophylactic agents to mitigate these side effects (McCann, Maguire, Miller, & Kearney, 2009). With respect to disease-related symptoms, such as tumor-related pain, effective pain management approaches are essential to maintain or improve QOL, and palliative treatment to reduce tumor burden may also be effective (Chih et al., 2012). In addition, patients should be regularly monitored for depression and other emotional disturbances and provided with effective medication when warranted.

## Other Supportive Approaches

Individualized, comprehensive patient education provided by the advanced oncology practitioner is one of the most effective forms of supportive care. By increasing their knowledge of the disease and its management, patients with MBC can gain a better understanding of what to expect from their treatment, including the associated side effects. Knowing that there are available measures to address concerns such as effective side-effect management has the potential to improve the psychological and emotional well-being of patients. In addition, educated patients can more effectively partner with their health-care providers in recognizing and reporting treatment- and disease-related issues, thereby enabling earlier interventions. Open communication between advanced oncology practitioners and educated patients can also help ensure that patients’ preferences are made known, which may positively affect treatment adherence. Table 2 lists strategies for promoting treatment adherence through a variety of approaches.

**Table 2:**
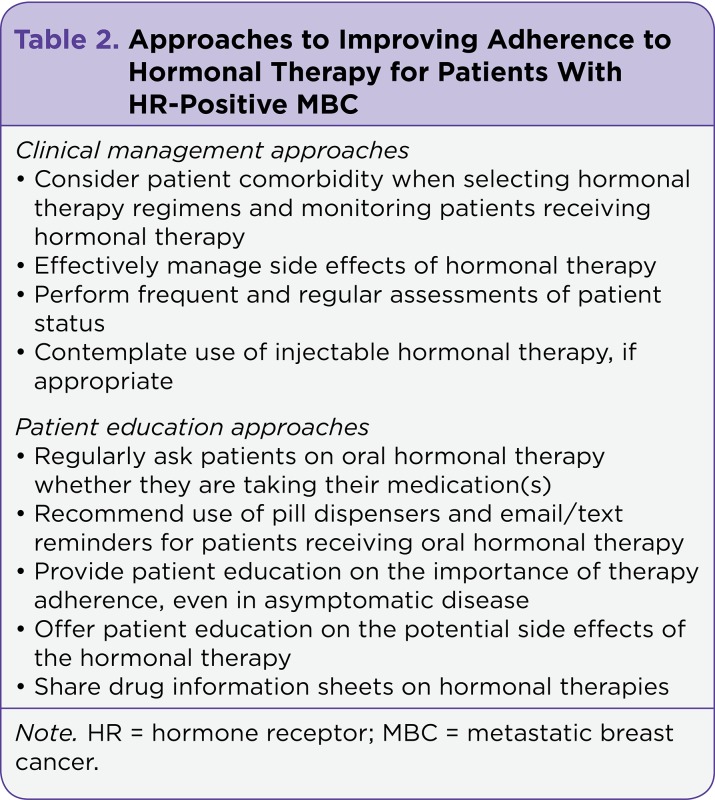
Approaches to Improving Adherence to Hormonal Therapy for Patients With HR-Positive MBC

In addition to clinical assessments, advanced oncology practitioners should routinely assess their patients’ concerns (including the financial impact of care) and psychological status and respond to the emotional concerns of their patients with MBC (Aranda et al., 2006; Halkett et al., 2006). There is evidence that addressing end-of-life issues may be particularly effective when applied early in the course of treatment. In one study, a discussion-based palliative care intervention focusing on the benefits of hospice and information regarding living wills and advanced directives delivered by nurse practitioners was instituted for patients with advanced cancer (Dyar, Lesperance, Shannon, Sloan, & Colon-Otero, 2012). Hospice knowledge and QOL were measured among intervention and control group study participants. Improvements in both emotional and mental QOL were statistically significant among patients randomized to receive the intervention vs. the control group.

## Conclusion

Many patients with HR-positive MBC respond to the sequential administration of hormonal therapies and are able to postpone receiving cytotoxic therapy. An understanding of the mechanisms of action and side-effect profiles of the different hormonal agents is important when selecting optimal treatment and effectively managing patients receiving these therapies. The role of the advanced oncology practitioner in the management of patients with HR-positive MBC is central, and the establishment of open communication with educated patients is essential. Although MBC is considered to be incurable, the effective implementation of a wide range of treatment and supportive approaches has the potential to positively impact the QOL and survival of patients with HR-positive MBC.

## Acknowledgments

Dr. Visovsky would like to thank Susan Moench, SCI Scientific Communications & Information, for her editorial support funded by AstraZeneca LP.
